# A Comparison of Clinical, Electro-Diagnostic, Laboratory, and Treatment Outcome Differences in a Cohort of HIV-Infected and HIV-Uninfected Patients With Myasthenia Gravis

**DOI:** 10.3389/fneur.2021.738813

**Published:** 2021-10-15

**Authors:** Kaminie Moodley, Pierre L. A. Bill, Vinod B. Patel

**Affiliations:** Department of Neurology, University of KwaZulu-Natal, Durban, South Africa

**Keywords:** HIV, AIDS, Myasthenia Gravis, IV cyclophosphamide (CY), Plasma exchange (PLEX, PE), intravenous immunoglobulin (IVIG)

## Abstract

There is limited literature comparing the clinical parameters and treatment outcomes in HIV-infected and HIV-uninfected myasthenia gravis (MG) patients. The aim of the study was to investigate the clinical differences and treatment outcomes in the two categories of patients, particularly the safe use of immunosuppressive therapy in immunocompromised patients. The study was a retrospective analysis of medical records of MG patients from the neuromuscular unit at Inkosi Albert Luthuli Central Hospital in Kwa-Zulu Natal between 2003 and 2019. One hundred and seventy-eight (178) patients fulfilled the clinical criteria for MG. Twenty-four (13.4%) were HIV-infected and 154 (86.5%) were HIV-uninfected. There were 116 (65%) females, median 45 years, (IQR 40–62), 90 (50.5%) black African, 66 (37%) Indian, 20 (11.2%) white, and 2 (1.1%) of mixed ancestry. In the HIV-infected cohort, 20 (87%) had generalized MG, 12 (50%) bulbar, and 14 (60.9%) respiratory onset MG, 12 (50%) presented with MG Foundation of America (MGFA) class five diseases at diagnosis, six (25%) presented with MG crisis during the 5-year follow-up. Thirteen (54%) of the HIV-infected group required rescue therapy using (plasma exchange or IV immunoglobulin) combined with pulse cyclophosphamide compared with 17 (11%) in the HIV-uninfected cohort, respectively. At 5 years, 8 (33%) of the HIV-infected group remained refractory to treatment compared with 10 (6.5%) HIV-uninfected cohort, respectively. No adverse events were documented in HIV-infected patients receiving combination rescue therapy (PLEX or IVIG combined with IV cyclophosphamide). In conclusion HIV-infected MG patients are more likely to require combination rescue therapy with PE/IVIG and IV cyclophosphamide compared with those who were HIV-uninfected. No side effects were documented in the HIV-infected group receiving the above therapy.

## Introduction and Background

Myasthenia gravis (MG) is an autoimmune disease of the neuromuscular junction, with a reported prevalence of 150–250 cases per million individuals and an estimated annual incidence of 8–10 cases per million person years (1, 2). This incidence, including South Africa, is similar worldwide (3).

In South Africa, there are eight million people living with human immunodeficiency virus (HIV) infection (4). The coexistence of HIV infection with MG occurs uncommonly. The etiological association between MG and HIV is uncertain. HIV may precede, be coincident, or complicate MG. However, the management of MG in the setting of HIV infection is uncertain as data are limited to a small case series from South Africa and case reports (5–19).

The use of immunosuppressant drugs including azathioprine and corticosteroids in HIV-infected patients with other neuromuscular diseases has been previously described (20–22). However, the use of rescue therapy, with intravenous immunoglobulin (IVIG), plasma exchange (PLEX), or pulse IV (intravenous) cyclophosphamide for poorly controlled MG in the setting of HIV has been described in case reports only (9, 10, 23–26). The use of these agents especially IV cyclophosphamide is of concern in patients who are HIV-infected as they are at risk for opportunistic infections especially those with low CD4 counts. Tuberculosis (TB) is endemic in South Africa and the prevalence has doubled in the HIV era (27). Other opportunistic infections such as candidiasis, aspergillosis, herpes simplex, herpes zoster, and toxoplasmosis are additional infective risks.

We aimed to describe and compare the clinical, demographic features, response to immunosuppressant therapy, in particular PE/IVIG and IV cyclophosphamide in the HIV-infected MG category with the HIV-uninfected MG category.

## Methods

The study was a retrospective chart review of a cohort of patients with MG from the neuromuscular clinic at Inkosi Albert Luthuli Central Hospital (IALCH) in Durban between 2003 and 2019. The study was approved by the University of KwaZulu-Natal (UKZN) Biomedical Research Ethics Committee (ethics number: BE 272/15). This unit provides a service to ~11 million people. An estimated 19% of the South African population is HIV positive in the 15–49 years age category. The province of Kwa-Zulu Natal (KZN) has 40% of the HIV burden in SA (4).

Patients fulfilling the clinical criteria for MG (fatigable, fluctuating weakness of the limb, face, neck, eyelid or ocular movements, dysarthria, dysphagia) with one or more positive confirmatory tests from the following two categories: (a) AChR-antibody serology and (b) neuromuscular junction dysfunction as evidenced by a positive repetitive nerve stimulation test (RNS) showing a decrement of >10%, positive neostigmine, or edrophonium test, and strongly positive ice pack test. If serology for AChR Ab was negative, neuromuscular junction disorders such as LEMS, botulism, and other neurological diseases were excluded clinically and electrophysiologically (28) (see Japanese guidelines).

Patients were excluded if they did not meet the clinical criteria for MG, or the HIV status was unknown, insufficient data were available to adequately assess functional scales, or they were lost to follow-up. Data extracted included demographic features, duration, onset, and course of the disease, clinical presentation, and drug therapy including antiretroviral therapy (ART) in the HIV-infected category. Response to therapy (number of exacerbations and time to remission), functional recovery scored as Manual Muscle Testing (MMT) (29), MG quality of life (MG-QOL-15) scale (30), and MG activity of daily living (ADL) (31) were extracted pre-treatment and post treatment at 3 to 6 monthly intervals. Adverse events to treatment including opportunistic infections were recorded. Electrophysiological data (RNS), blood tests including AChR-Ab, CD4 counts, and viral loads were available, CT chest, and histology of the thymus if available were included in the analysis. The tests for anti-MuSK, agrin, LRP4 antibodies were not available in South Africa.

Our current management protocol for mild to moderate MG (including ocular MG), regardless of HIV status is anticholinesterase inhibitors (pyridostigmine) combined with corticosteroids and/or azathioprine as first-line therapy. Other immunosuppressant therapy (including IVIG, rituximab, and cyclophosphamide) are added if patients were refractory or developed side effects to first line therapy. Patients with myasthenic crisis received PLEX or IVIG. Cyclophosphamide was reserved for patients in crisis, refractory to PLEX or IVIG. Four to six doses of intravenous cyclophosphamide were given at 2 weekly or monthly intervals at a dose of 500–1,000 mg combined with 2-mercaptoethane. Patients were adequately counseled regarding side effects of treatment and informed consent was obtained. The dose and frequency was adjusted at the discretion of the clinician depending on the clinical response and adverse events such as leukopenia, thrombocytopenia, or anemia (neutrophil count of <1,000 cells/μl, lymphocyte count <500 cells/μl, platelet count <50 cells/μl, or hemoglobin <8 g/dl, respectively) or complicating infections. All patients were screened for existing infection with a full blood count and differential white blood count, urine microscopy, and culture, and chest X- ray. They were screened for hepatitis B and C prior to being prescribed methotrexate or rituximab. Maintenance therapy, if required, included pyridostigmine, corticosteroids, used individually or in combination depending on individual patient requirements.

**Figure 1:**
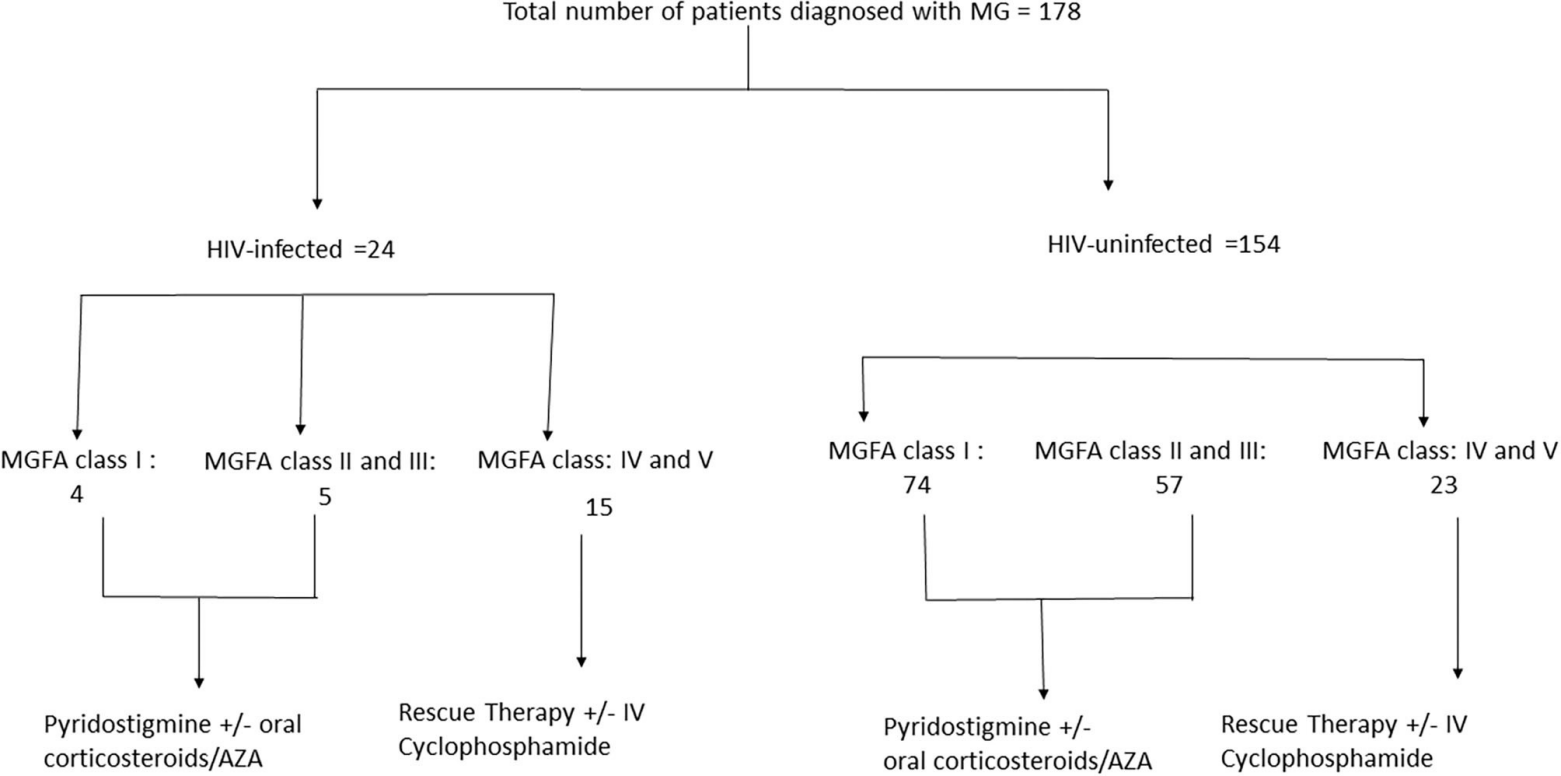
Consort diagram.

The cohort was divided into two categories of HIV-infected MG and HIV-uninfected MG. Within each category patients were further classified according to severity at presentation (see Figure 1). Data at 1 month, 6 months, 1 year, 3 years and 5 years time points were reviewed. The definitions as per Myasthenia Gravis Foundation of America (MGFA) post-interventional classification (32–35) used in this study are: (a) minimal manifestation status MMS 0–3, (b) complete stable remission (CSR), (c) pharmacological remission (PR), and (d) exacerbation (34). MG crisis was defined as life-threatening rapid worsening of MG with potential airway compromise from ventilatory or bulbar dysfunction (35). Refractory MG was defined as post interventional status of the affected patient being unchanged or worse after corticosteroids and at least two other immunosuppressive agents (AZA, MTX, and MMF), which are used individually or in combination in adequate doses for at least 6 months with persistent symptoms or side effects that limit functioning (35). Time to remission was defined as time to reach CSR, PR, or MMS-(0–3).

In this article combination rescue therapy refers to PE/IVIG with IV cyclophosphamide.

### Data Availability

Anonymized data will be shared by request from any qualified investigator.

### Statistics

Characteristics associated with HIV-infected and HIV-uninfected MG patients were compared using Chi Square tests for categorical variables. Functional scores of ADL, QOL, and MMT were initially categorized as positive (>0) and negative (0). Fisher's exact test was used to compare HIV-infected and HIV-uninfected patients at each time point. Overall functional scores were summarized by medians (IQR) and Wilcoxon's rank-sum test used for comparisons at each time point. Because of small numbers, geometric means were used for patients receiving combination therapy (PLEX/IVIG + IV cyclophosphamide) and *t*-tests used for comparison. A random effects model including HIV status and time point adjustments for repeated measures was then used to examine trends over time between the two groups. A *z*-test was used to compare groups and time points. To compare the rate of change, the model was run separately for each group and the beta coefficient for time compared. A Kaplan–Meier (KM) curve was subsequently used to determine if time to remission differed between groups. Follow-up time was used to censor patients not obtaining remission (CSR, PR, and MMS 0–3), and truncated at 60 months. A log rank test was used to compare the remission curves between groups. Chi-squared tests were used in order to determine if confounders such as demographic factors, and clinical characteristics (age, race, gender, antibody status, thymic pathology) associated with HIV status influenced outcome. These factors were examined in the group receiving cyclophosphamide, and only race was found to be significantly different. Medians of MGADL, MGQOL, and MMT score were compared between HIV-infected and HIV-uninfected patients receiving IV Cyclophosphamide at diagnosis, 3 and 5 years using Wilcoxon's rank-sum test. Since all HIV-infected patients receiving cyclophosphamide were black African, the analysis was repeated for black patients only. Stata/IC V1.5 was used for statistical analysis.

## Results

One hundred and seventy-eight (178) patients fulfilled the clinical criteria for MG of which 116 (65%) were females. The demographic characteristics of the cohort, including gender, age, and ethnic distribution are detailed in Table 1. Twenty-four (13.4%) were HIV-infected. Notable findings in the HIV-infected myasthenia gravis group of patients were a younger median age and a predominance of black females. HIV infection preceded the development of myasthenia gravis in the majority of HIV infected patients. One patient (4%) became HIV-infected several months after MG was diagnosed. Viral loads and CD4 counts are listed in Table 1. Thirteen (54.2%) of the HIV-infected cohort were on efavirenz, tenofovir, and emtricitabine before the diagnosis of MG. Six patients on ART had undetectable viral loads. Ten (41.8%) patients were diagnosed with HIV-infection at the time of MG and were later commenced on ART. Six (60%) patients received ART while in ICU and four (40%) a month after discharge.

**Table 1:** Demographic characteristics of HIV-infected and HIV-uninfected patients with MG.

	**HIV-infected**	**HIV-uninfected**	***P*-value**
	**(*n* = 24)**	**(*n* = 154)**	
**Age (median, IQR)**	34 (27.5–41)	47.11 (32–65)	0.001
Female	19 (79.2)	97 (63)	0.12
**Race (%)**			<0.001
Black	23(95.8)	67(43.5)	
Indian	1 (4.2)	65 (42.2)	
White	0 (0)	20 (13)	
Mixed Ancestry	0 (0)	2(1.3)	
**Autoimmune disease**			<0.001
Diabetes Mellitus		62 (40)	
RA		2 (1.3)	
LEMS	1 (4)	0 (0)	
Dermatomyositis		2 (1.3)	
Hashimoto's thyroiditis		6 (3.9)	
**Time of diagnosis of MG in relation to HIV infection (%)**			
After HIV	13 (54.2)		
At the same time as HIV	10 (41.6)		
Before HIV	1 (4.2)		
CD4 count at diagnosis (cells/μl) (median, IQR)	190 (186–361)		
Viral Load at diagnosis (copies/ml) (median, IQR)	17,222 (9,154–98,100)		

*LEMS, Lambert Eaton Myasthenic Syndrome; RA, Rheumatoid arthritis; HIV, human immunodeficiency syndrome; MG, myasthenia gravis*.

Sixty-two (40%) HIV-uninfected patients had T2 diabetes mellitus (T2 DM), and 10 had other autoimmune disorders, whereas only one HIV-infected patient had Lambert–Eaton myasthenic syndrome (LEMS). The diagnosis of combined LEMS and MG was based on clinical findings, positive high-frequency repetitive stimulation studies, and positive AChR-Ab, and voltage-gated calcium channel antibody (36).

Clinical findings at diagnosis are listed in Table 2. Bulbar, respiratory, and limb muscle weakness was more severe in the HIV-infected MG group, while signs of ocular muscle weakness was more common in the HIV-uninfected MG cohort. The HIV-infected MG cohort, which was more severely affected, with 12/24 patients (50%) requiring ventilation at presentation and exacerbations, including MG crisis, were more common. The most common precipitating factor documented in the HIV-uninfected group was preceding infection (upper respiratory tract, urinary tract infection), and in the HIV-infected group, most often no identifiable factor was identified.

**Table 2:** Clinical Profile of HIV-infected and HIV-uninfected patients with MG.

	**HIV- infected**	**HIV-uninfected**	***P*-value**
	**(*n* = 24)**	**(*n* = 154)**	
**Clinical presentation**			<0.001
Ocular muscle weakness	20 (83.3)	118 (76.6)	0.306
Bulbar muscle weakness	12 (50)	48 (31.1)	<0.001
Respiratory muscle weakness	14 (60.9)	26(16.9)	<0.001
Skeletal muscle weakness	21 (87.5)	80 (51%)	<0.001
**MGFA clinical class at presentation (%)**			
I	4(16.7)	74(48.1)	0.001
IIa/IIb	3 (12.5)	35(22.7)	
IIIa/IIIb	2 (8.2)	22(14.2)	
IVa/IVb	3 (12.5)	9 (5.8)	
V	12 (50)	14 (9.1)	<0.001
**Number of exacerbations during follow up**			0.02
<5	6 (25)	85 (55.6)	
5–10	10 (41.7)	65 (42.2)	
>10	8 (33.3)	4 (16.2)	
≥1 crises	6 (25)	7 (4.5)	
**Cause of exacerbations**			
Preceding infection	2 (UTI)	25 (UTI/URTI)	
Drug induced (antimicrobial therapy)	2	10	
Defaulted treatment	1	10	
Unknown	14	15	

*MG, Myasthenia Gravis; MGFA, Myasthenia Gravis foundation of America; UTI, urinary tract infection; URTI, Upper respiratory tract infection*.

Investigations, treatment, and outcome are listed in Table 3. Fifteen (62%) of the HIV-infected cohort were AChR-Ab positive compared with 124 (80.5%) of the HIV-uninfected cohort, *p* = 0.047. A greater number of HIV-infected patients had a decremental response (83%) on RNS compared with the HIV-uninfected category (52%). This may reflect more frequent generalized disease in the HIV-infected category.

**Table 3:** Investigations, treatment and clinical status at follow up in the HIV-infected and HIV-uninfected cohort.

	**HIV-infected**	**HIV-uninfected**	***P*-value**
	**(*n* = 24)**	**(*n* = 154)**	
**Investigations**			
Positive AchR Ab test (%)	15(62)	124 (80.5)	0.047
Positive ice pack test (%)	18 (75)	136 (87)	0.11
Positive edrophonium test (%)	24(100)	115 (74.6)	0.12
Decremental response of >10% on RNS test (%)	20 (83)	82 (52)	0.005
**CT thymus**			0.47
Normal	7 (29)	65 (42)	
Hyperplasia	12 (50)	62 (40)	
Mass lesion	5 (21)	27 (18)	
Thymectomy	20 (83)	97 (63)	0.27
**Histology**			
Thymic hyperplasia	15 (75)	73 (75)	
Thymoma	3 (15)	23 (23)	
Thymolipoma	1 (5)	1 (1)	
Thymic carcinoma	1 (5)	0 (0)	
Treatment			
**Rescue/induction therapy**			
IVIG only	1 (4.2)	2 (1.2)	
PE only	1 (4.2)	3 (2)	
PE or IVIG + IV cyclophosphamide	13 (54)	17 (11)	0.001
IV neostigmine	13 (54)	21 (14)	
**Maintenance therapy**			
AZA/oral pyridostigmine and prednisone	20 (84)	118 (77)	
Rituximab	0 (0)	1 (0.6)	
Oral pyridostigmine only	4 (16)	36 (24)	
Side effects of Immunosuppressive therapy			
Opportunistic infections	0 (0)	0 (0)	
Haemorrhagic cystitis	0 (0)	2 (11.7)	
**Clinical status at 5 years follow up**			
CSR	0 (0)	2 (1.3)	
PR	0 (0)	2 (1.3)	
Minimal manifestation status (0–3)	16 (64)	140 (90)	<0.001
MMS-0	0 (0)	58 (37,8)	
MMS-1	2 (9)	22 (14.2)	
MMS-2	6 (25)	15 (9.8)	
MMS-3	8 (33)	35 (22.7)	
Refractory disease	8 (33)	10 (6.5)	<0.001

*AChR Ab, Acetylcholine receptor antibodies; RNS, Repetitive nerve stimulation; IVIG, intravenous immunoglobulin; PE, plasma exchange; IV, intravenous; AZA, Azathioprine; MMS, minimal manifestation status; CSR, complete stable remission; PR, pharmacological remission*.

There were no significant differences between the two cohorts with respect to the radiological and histological findings of the thymus. Eighty three percent (83%) of the HIV-infected patients, at a median age of 32 years, IQR (26–38), and 63% of the HIV-uninfected patients at a median age of 44 years, IQR (30–48) had a thymectomy performed. Patients were stabilized on IVIG/PE with/without IV cyclophosphamide prior to the thymectomy.

Four highly refractory patients in the HIV-uninfected category and three in the HIV-infected category had a thymectomy during the crisis. They had received PLEX and IV cyclophosphamide during the crisis and post thymectomy. In both categories, patients improved 2–4 weeks post-thymectomy. There was no difference in the outcome between the two categories of patients.

In the HIV-infected MG cohort on PLEX/IVG and IV cyclophosphamide, the median CD4 count at diagnosis was 110 cells/μl (IQR 96–193 cells/μl) and 88 cells/μl (IQR 68–108 cells/μl) 2 weeks after cyclophosphamide. The median viral load at diagnosis was 47,481 copies/ml (IQR 45,545–119,292 copies/ml). HIV viral loads were not repeated after cyclophosphamide unless indicated.

With regard to treatment, 13/24 (54%) of the HIV-infected MG group required rescue therapy using either PE or IVG combined with pulse IV cyclophosphamide compared with 17/154 (11%) in the HIV-uninfected cohort. Maintenance therapy was not significantly different between the two groups.

At 5 years, after correcting for baseline severity of disease, eight (33%) of the HIV-infected group remained refractory to treatment and 16 (64 %) obtained remission compared with 10 (6.5%) and 144 (93 %) in the HIV-uninfected cohort, respectively.

**Figure 2:**
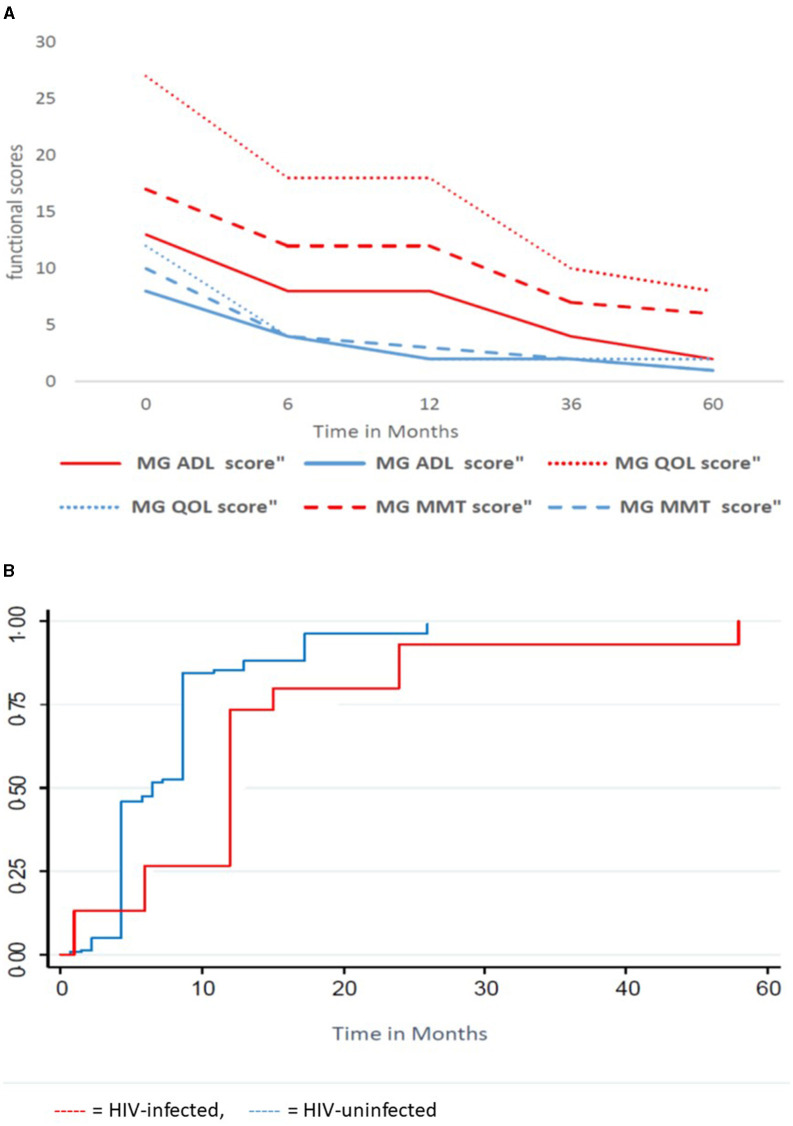
Functional scores and time to remission for the HIV-infected and HIV-uninfected cohort. **(A)** Functional Scores over time, **(B)** Kaplan Meier Curve of survivor function. MMT, Manual Muscle Testing; ADL, Activity of daily living, QOL, Quality of life; 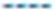, HIV-uninfected; 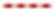, HIV-infected.

Figure 2 shows functional scores and time to remission over 60 months in the entire HIV-infected and HIV-uninfected cohort. The median MGADL, MMT, MGQOL (Figure 2A) functional scores are significantly higher in the HIV-infected cohort, *p* < 0.001, compared with the HIV-uninfected cohort. The above scores decrease significantly over time with no statistical difference in the rate of decrease between cohorts. Figure 2B shows the KM curve for the entire cohort. One hundred and sixty (89.8%) of 178 patients obtained remission over a median of 72 months (IQR 48–126 months). Within a median time of 9 months (95% CI: 6–12 months), 144 of 155 (93.6%) HIV-uninfected patients obtained remission, while 16/23 (64%) of the HIV-infected group obtained remission in a median time of 12 months (95% CI 6–15 months). The probability of obtaining remission was significantly greater in HIV-uninfected cohort compared with HIV-infected cohort (OR 7.25; 95% CI: 2.5–21.0), *p* < 0.001. The Kaplan–Meier curves of survivor function differed significantly by HIV status, *p* = 0.0007 (log rank test of equality of survivor function). This indicates that the time to obtaining remission was significantly shorter for HIV-uninfected patients than HIV-infected patients.

**Figure 3:**
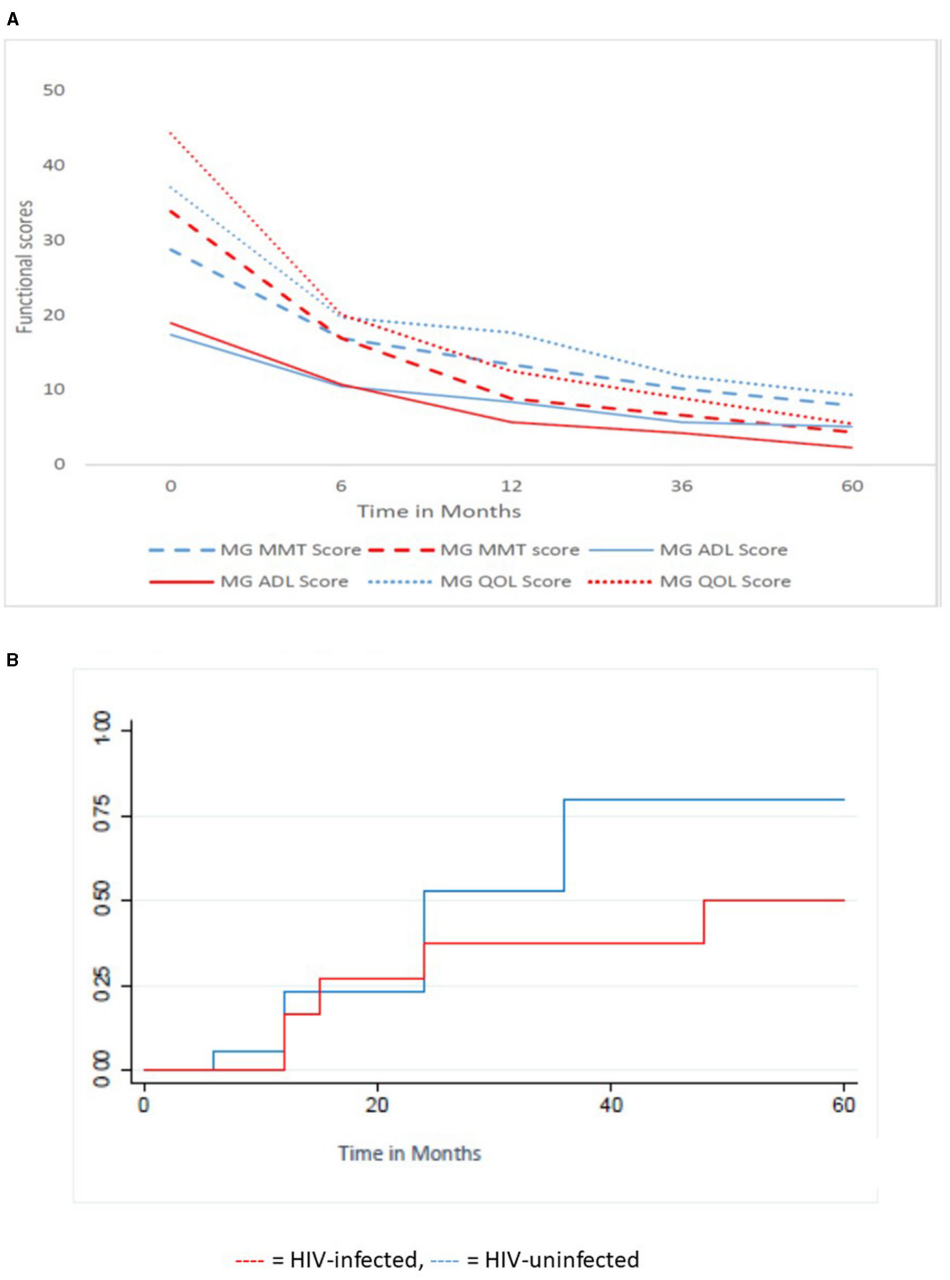
Functional scores and Kaplan Meier time to remission for the HIV-infected (13 patients) and HIV-uninfected (17 patients) cohort receiving PLEX/IVIG and IVI cyclophosphamide. **(A)** Functional scores over time, **(B)** Kaplan Meier Curve of Survivor function. MMT, Manual Muscle Testing; ADL, Activity of daily living; QOL, Quality of life; 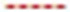, HIV-infected; 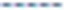, HIV-uninfected.

Figure 3 shows the functional scores and time to remission in the HIV-infected MG and the HIV-uninfected MG receiving PLEX/IVIG and IV cyclophosphamide. The mean MMT, MG-QOL, MGADL (Figure 3A) scores are significantly higher in HIV-infected MG compared with HIV-uninfected MG. Over 5 years, the scores decrease significantly, the rate of decrease is significantly greater in the HIV-uninfected MG compared with HIV-infected MG (*p* = 0.0014, 0.0018, 0.001, respectively). Figure 3B reflects the KM curve for those who received IVIG or PLEX with cyclophosphamide. HIV-uninfected patients (median follow-up 72 months, IQR: 48–192 months) had a similar follow-up time to HIV-infected patients (median follow up 60 months, IQR 12–72 months), *p* = 0.08. Of the 30 patients on the above combination therapy, 13/17 (76.5%) in the HIV-uninfected category obtained remission compared with 5/13 (38.7%) in the HIV-infected category (OR 4.3, 95%; CI: 0.7–30.6) but this did not reach statistical significance, *p* = 0.13. The median time to remission for the 13 HIV-uninfected patients was 15 months (IQR 12–36 months) and for the five HIV-infected patients was 24 months. This was not statistically significant (*p* = 0.68).

Confounding factors such as age, gender, thymic pathology, and antibody status were similar in both cohorts receiving IV cyclophosphamide except for race. Since all HIV-infected patients receiving cyclophosphamide were black African, the analysis was restricted to black patients only. However, there were no significant differences in outcome in the black only groups.

No adverse events related to therapy were documented in patients receiving combination rescue therapy (PLEX or IVIG + IV cyclophosphamide) in at least the first 6 weeks post-therapy, in particular there were no opportunistic infections in the HIV-infected category during this time period and during the rest of follow-up. In the HIV-uninfected category, two patients experienced hemorrhagic cystitis.

## Discussion

In the above study, significant differences are that (a) HIV-infected MG patients were more likely to be young, black females. This is consistent as MG is more common among young females and that HIV is more prevalent among young black South African females, (b) HIV-infected MG presented with more severe generalized disease, as evidenced by MGFA clinical grades and functional scores (MMT, MGQOL, MGADL), and was more likely to present with bulbar-respiratory failure requiring ventilation. This category of patients were managed following the treatment protocol for HIV-uninfected MG patients which includes, respiratory-bulbar support, rescue therapy using either IVIG or PLEX, and IV cyclophosphamide if required, followed by maintenance therapy. The use of a neostigmine infusion in patients admitted to the ICU or a high care setting was useful for symptomatic control of the disease, (c) the rate of functional improvement was significantly lower, time to remission was longer, and response to therapy (number of exacerbation or crises) was higher in the HIV-infected cohort compared with the HIV-uninfected cohort. Time to remission and side effect profile for the group on combination rescue therapy (IV cyclophosphamide/PLEX/IVIG) were not different in the two categories. Patients with milder disease were managed with oral immunosuppressant therapy (AZA and corticosteroids and pyridostigmine) with similar outcomes in both categories.

The safe use of IV cyclophosphamide is not well-described in the setting of HIV-infected MG. Clinical guidelines on safe and effective prescription of ARTs with concomitant cytotoxic immunosuppressive agents is limited, except in the setting of AIDS-related B-cell lymphoma (37, 38). Bone marrow reserve, drug–drug interactions with ART, prophylaxis for mycobacterium, fungal, and viral infections, and the use of granulocyte-colony-stimulating factor when complicated by bone marrow suppression are all considerations in the management of HIV-infected patients with MG. Standard treatment protocols from Haem-oncology centers treating AIDS related B cell-lymphoma may lend guidance. This study suggests that using the protocol listed in the Methods section of the article, regardless of the CD4 count or viral load, may be safe, provided patients are carefully monitored. None of the patients received prophylaxis for opportunistic infections or granulocyte-stimulating factor. As expected, CD4 counts decreased following cyclophosphamide administration with no documented clinical infection. If severe neutropenia was observed, IV cyclophosphamide was withheld until the neutropenia recovered spontaneously. The use of prophylactic drugs for opportunistic infections or granulocyte stimulating factor may be of value in this setting. Despite newer immunosuppressive therapy becoming available, IV cyclophosphamide is an easily available and effective agent, especially in a resource with limited center, provided patients are carefully monitored and adequately counseled regarding adverse events.

Various autoimmune diseases occur between acquisition of HIV infection and clinical AIDS. These include systemic lupus erythematosus, rheumatoid arthritis, idiopathic thrombocytopenic purpura, and neurological conditions such as AIDP, CIDP, and inflammatory myositis (24). The mechanisms resulting in autoimmune disease in HIV infected individuals are poorly understood, although B lymphocyte dysfunction and molecular mimicry between HIV proteins and autoantigens may play a role.

MG, including anti-MuSK related MG, occurring during immune reconstitution has been reported (6, 13, 39, 40). Ten patients in our cohort who were commenced on ARTs after the diagnosis of MG showed transient worsening of their MG in the initial stages of commencing ARVs. This is supported by similar findings reported by Heckmann et al. (10). This worsening may coincide with the first wave of immune reconstitution. Several immune factors may act in synergy during immune restoration resulting in exacerbations. Factors include pathogenic T-cell response derived from the memory T-cell pool, a Th1-type CD4 and CD8 response, loss of function of T regulatory cells, and overproduction of IL2 and IL6. This may result in expansion of the autoreactive repertoire and immune aggression (41, 42). Whether long-term ART prevents MG in HIV is unknown, as 13 patients developed MG while on ART (6).

Loss of central tolerance, which is pathognomonic of MG, may occur in HIV. Tropism of HIV to the thymus and antigenic mimicry between normal thymic components and core p17 and p24 protein of HIV have been described (43). McCune reported that the receptors for HIV are present on 90% of all thymocytes and intrathymic macrophages. Although theoretically thymic atrophy is expected in HIV, 75% of the HIV-infected cohort had thymic hyperplasia, 5% thymoma, thymolipoma, and thymic carcinoma. One may speculate that with HIV infection, chronic peripheral T-cell lymphopenia (median CD4 count 190) may result in thymic rebound and upregulation of thymopoeisis. However, the effect of thymectomy on the immune profile in HIV and whether the thymus contributes to the pathogenesis of MG is unknown. The above will be better explored in a prospective study.

In the above cohort, the HIV-infected MG patients with severe bulbar-respiratory failure were more likely to be AChR-Ab negative or have a very low titer of AChR-Ab (26). This may support an anti-MuSK pathogenesis or an unknown antibody compared with those with antibodies to AChR, agrin, or LRP4 (44–46). Future testing for MuSK antibodies or identifying a new antibody will be useful. Specific antibody testing when using a broad immunosuppressant such as cyclophosphamide is irrelevant until more specific therapies are available. Future studies, which include antibody panels and targeted use of monoclonal antibodies such Rituximab in HIV is required. One HIV-uninfected patient with refractory MG received Rituximab with good results. The use of Rituximab in HIV-infected MG is limited to case reports (10, 47). Kuntzer described successful use of Rituximab in an HIV-infected patient with immune reconstitution bulbar onset MuSK MG (47).

Limitations of the study are that it is a retrospective, hospital-based study, and therefore, patients may have been missed, and results may not be generalizable to the community. Furthermore, serological testing for MG was incomplete as the tests for anti-MuSK, agrin, LRP4 antibodies are not available in South Africa. One can only draw broad general conclusions regarding the outcome and side effect profile in the two groups due to the discrepancy in numbers, which makes statistical comparison difficult. More symmetrical sample sizes with randomized controlled prospective studies are needed to measure reliable response to therapy and properly assess the safety profile of IV cyclophosphamide in HIV-infected patients with respect to infections and bone marrow suppression. However, this is not possible due to the low frequency of myasthenia gravis in the context of HIV. No infections attributed to recent use of IV cyclophosphamide were documented in the HIV-infected group despite regular follow-up and screening. This may reflect a type 2 statistical error as it is possible that patients with minor infections were not reported or were managed elsewhere. Confounding factors were not found to be significantly different between the HIV-infected and HIV-uninfected groups except for race. Hence, the analysis of outcome was adjusted for race only, and multivariate analysis was deemed unnecessary. Despite the above limitations, the study reflects the real-life experience of managing patients living with MG and HIV in the South African context.

## Conclusion

HIV-infected MG patients present with more severe bulbar-respiratory signs requiring supportive care in ICU. This study suggests that immunosuppressive drugs, including IV cyclophosphamide may be safe and efficacious in HIV-infected MG. Prospective studies of MuSK or new antibodies, immune function studies, and possibly thymic histology may be valuable to explain the pathogenesis of MG in HIV and perhaps segregate alternate therapeutic avenues. This will allow for the establishment of treatment guidelines of MG in HIV.

## Data Availability Statement

The raw data supporting the conclusions of this article will be made available by the authors, without undue reservation.

## Ethics Statement

The studies involving human participants were reviewed and approved by UKZN Biomedical Research Ethics Committee. Written informed consent for participation was not required for this study in accordance with the national legislation and the institutional requirements.

## Author Contributions

KM developed the concept, collected and analysed the data and generated the manuscript, VP helped develop the concept and reviewed the manuscript and PB reviewed the manuscript. All authors contributed to the article and approved the submitted version.

## Funding

This work was supported by the personal payment from research funds of PB.

## Conflict of Interest

The authors declare that the research was conducted in the absence of any commercial or financial relationships that could be construed as a potential conflict of interest.

## Publisher's Note

All claims expressed in this article are solely those of the authors and do not necessarily represent those of their affiliated organizations, or those of the publisher, the editors and the reviewers. Any product that may be evaluated in this article, or claim that may be made by its manufacturer, is not guaranteed or endorsed by the publisher.
